# Treatment of Bisphenol A-Containing Effluents from Aerobic Granular Sludge Reactors with the Use of Microfiltration and Ultrafiltration Ceramic Membranes

**DOI:** 10.1007/s11270-017-3450-1

**Published:** 2017-07-15

**Authors:** M. Zielińska, A. Cydzik-Kwiatkowska, K. Bułkowska, K. Bernat, I. Wojnowska-Baryła

**Affiliations:** Department of Environmental Biotechnology, University of Warmia and Mazury in Olsztyn, Słoneczna Str. 45G, 10-709 Olsztyn, Poland

**Keywords:** Bisphenol A, Aerobic granules, Secondary effluent, Microfiltration, Ultrafiltration

## Abstract

This study investigated the use of ceramic membranes to remove total suspended solids (TSS), organics (expressed by chemical oxygen demand, COD), and bisphenol A (BPA) via microfiltration (MF, pore size 0.45 μm) and ultrafiltration (UF, *cutoff* 150 kDa) in post-treatment of effluents from aerobic granular sludge reactors (GSBRs). The efficiency of removal of COD, BPA, and TSS in MF was similar to that in UF; however, it was achieved at a lower pressure, which reduces energy consumption during the filtration process. Despite the similar quality of the permeates in MF and UF, the permeate flux averaged almost 20% higher in UF than in MF. The rejection coefficients were 77–82% for COD and 48–100% for BPA. In both MF and UF, TSS were totally removed. In the integrated system of aerobic granular sludge reactor and membrane installation, total removal of COD was 92–95% and that of BPA was above 98%, independently of the membrane technique. The high efficiency of BPA removal in MF and UF, despite pore sizes in the MF and UF membranes larger than the BPA molecules, suggests that some part of the BPA was first bound by particulate organic matter in the biologically treated wastewater before this sorbed form was removed by the membranes. Furthermore, the high removal of COD and BPA, even in MF, was attributed to adsorption on the membranes, in addition to sieve retention.

## Introduction

Bisphenol A (2,2-bis-4-hydroxyphenylpropane, BPA) is a xeno-estrogen that is widely used in the production of polycarbonate plastics, epoxy resins, etc. The volume of the world BPA production is predicted to surpass the 5.4 million t mark by 2015 (Merchant Research and Consulting 2014). This environmental pollutant is an endocrine disrupting compound (EDC) with relatively high biological activity. The most common source of BPA in the environment is wastewater. Although BPA can be degraded by microorganisms, it is hard to completely remove it from wastewater with conventional biological treatment methods. As a result, residual BPA is present in the effluents from municipal wastewater treatment plants in concentrations that can be up to 10,000 times higher than those that are estrogenic (Tanaka et al. 2000; Kasprzyk-Hordern et al. 2009). One of the reasons that biological treatment is not completely effective is because BPA can be sorbed on biofilm or suspended solids (Stringfellow and Alvarez-Cohen 1999). The suspended solids concentration in the effluent from granular sludge reactors is particularly high because the short sedimentation time is applied as a strong selective pressure for granulation (McSwain et al. 2005). Thus, post-treatment is necessary to lower the concentration of BPA in effluent from these systems.

For this purpose, membrane systems have recently become an important option. Although membrane filtration transfers BPA from one medium to another, which requires further treatment or disposal (Liang et al. 2015), membranes have a high retention capacity, which allows them to produce effluents with low concentrations of organic compounds (Liang et al. 2015). Membrane technology has progressed, reducing the costs involved (Nicolaisen 2002). Ceramic membranes have advantages over commonly used polymeric membranes: good thermal and chemical stability and high resistance to corrosion, abrasion, and fouling. These advantages allow highly efficient backwashing and make ceramic membranes more durable (Baker 2000). Ceramic membranes can also have much higher flux than polymer membranes because of weaker bonding between the foulants and the membranes (Lee et al. 2013). Thus, although ceramic membranes have higher initial costs, their advantages make them more competitive with polymeric membranes over the long term.

BPA rejection by membranes ranges widely, from 18% (Kimura et al. 2004) to >99.9% (Agenson et al. 2003), due to a strong relationship between rejection rate and membrane type; for phenolic compounds like BPA, the relationship between rejection efficiency and molecular weight *cutoff* is linear (Jung et al. 2007). Of the various options for membrane treatment of BPA-contaminated wastewater, microfiltration (MF) and ultrafiltration (UF) are less effective than nanofiltration, but they save costs because they can be operated at lower pressures, which makes them worth investigating with the aim of improving their efficiency. Although the pore sizes of MF and UF membranes are considerably greater than the size of the BPA molecules, they have achieved notable results in BPA retention due to the adsorption of BPA on suspended solids (Gómez et al. 2007). If most BPA is adsorbed to these solids, MF and UF filters can serve as secondary clarifiers and separate secondary effluent and BPA that is sorbed on suspended solids. This was confirmed by the study by Bing-zhi et al. (2010) who obtained 20–95% of BPA removal from drinking water in the MF system. Similarly, UF of 100 μg BPA/L with membranes of pore sizes of 10, 6, and 2 kDa gave the rejection efficiencies of 93.0, 88.9, and 97.7%, respectively (Dong et al. 2008).

The use of only membrane filtration for wastewater purification is limited by the clogging of membranes with pollutants (Sun et al. 2015), which shortens the filtration cycle and lower membrane life. In this study, membrane filtration was used as a second step after biological treatment to delay the drop in removal efficiency and to lengthen membrane life.

There is a lack of studies on the combination of membrane filtration with biological treatment of BPA-containing effluents from granular sludge reactors. Thus, we compared MF and UF ceramic membranes for the removal of BPA from effluent from granular sludge reactors operated at a short sedimentation time. We also tested the susceptibility of the ceramic membranes to fouling. These results will help to determine the most effective combination of ceramic membrane selectivity and permeability for removal of BPA from biologically treated wastewater with low BPA concentration.

## Materials and Methods

### Characteristics of Feed Wastewater

The experiments were run with biologically treated wastewater from granular sequencing batch reactors (GSBRs) with a working volume of 3 L. The constantly aerated GSBRs were operated at a volumetric exchange rate of 50%/cycle, length of a cycle of 8 h, hydraulic retention time of 16 h, solids retention time of 46–56 days, a temperature of 20 ± 2 °C, a pH of 7.5–8.0, and at the concentrations of mixed liquor suspended solids in the reactors of about 11 g/L in which organic fraction accounted for about 30%. The GSBRs treated synthetic wastewater, in which the average chemical oxygen demand (COD), ammonium, and phosphorus concentrations were 445 ± 79.8, 46.7 ± 6.0, and 10.5 ± 0.8 mg/L, respectively. This wastewater was spiked with BPA to concentrations of 2 mg BPA/L (reactor R2), 6 mg BPA/L (reactor R3), and 12 mg BPA/L (reactor R4). In a reactor R1, there was no BPA in the influent. The efficiencies of COD removal and nitrification were 87.9 ± 3.5 and 86.5 ± 3.7% in R1. The efficiencies of COD removal, nitrification and BPA removal were 91.1 ± 3.3, 91.2 ± 2.6, and 97.4 ± 0.8% in R2; 92.2 ± 3.2, 89.3 ± 6.3, and 98.3 ± 0.7% in R3; and 94.7 ± 1.7, 91.3 ± 2.0, and 99.3 ± 0.8% in R4, respectively. In the effluents, the concentrations of BPA, COD, and total suspended solids (TSS) are as given in Table 1. Because of high concentrations of COD and suspended solids, these effluents were post-treated with membrane filtration.

**Table 1:** Characteristics of effluents from GSBRs R1–R4 that were post-treated

Reactor	R1	R2	R3	R4
TSS (mg/L)	180.4 ± 14.2	123.6 ± 26.5	142.9 ± 13.5	149.8 ± 22.6
COD (mg/L)	132.0 ± 9.6	123.6 ± 21.6	142.5 ± 39.9	143.8 ± 13.8
BPA (mg/L)	–	0.053 ± 0.006	0.101 ± 0.011	0.085 ± 0.009

### Membrane Installation

**Fig. 1:**
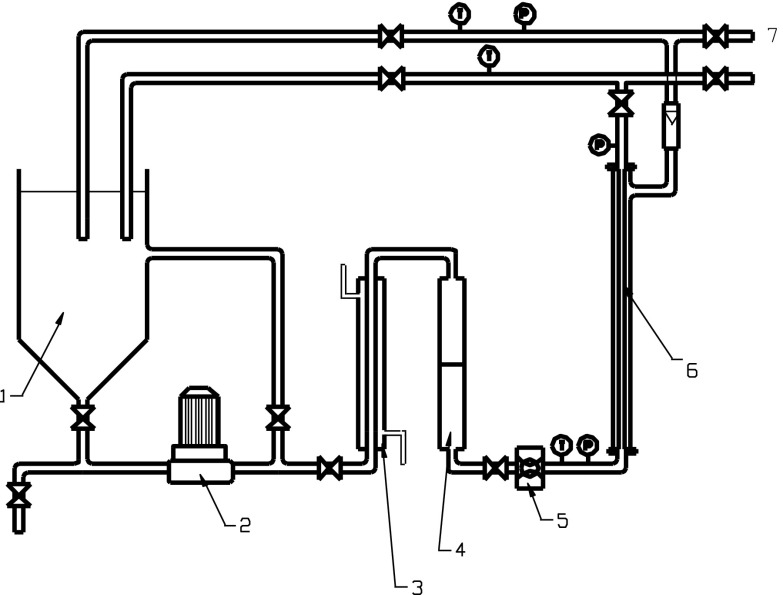
Scheme of the membrane installation: *1*—process tank, *2*—pump, *3*—heat exchanger, *4*—prefilter, *5*—flow control, *6*—membrane module, *7*—permeate sampling point, *T*—thermometer, *P*—manometer

The membrane installation consisted of a 10-L process tank, a high pressure pump (CRN(E), Grundfos), a membrane module placed outside the process tank, a flowmeter, a heat exchanger, a 1-mm prefilter, pressure gauges at the inlet and outlet of the membrane module, a line to circulate the retentate back to the process tank, and a line to receive permeate from the system (Fig. 1). The membrane module housed one Inside-Céram™ tubular asymmetric ceramic membrane (Tami Industries) that was made from a mixture of TiO_2_ and ZrO_2_. The membrane was 300 mm long with an external diameter of 25 mm and 23 channels of a hydraulic diameter of 3.5 mm inside. The total effective filtration area was 0.1 m^2^, and the specific area was 680 m^2^/m^3^. Ceramic membranes for MF (pore size 0.45 μm) and UF (*cutoff* 150 kDa) acted as secondary clarifiers and were used at pressures of 0.2 and 0.3 MPa, respectively. The membrane installation worked under *cross-flow* conditions. The feed solution was pumped into the membrane channels and the permeate came out from the external membrane walls. To limit membrane clogging, backwashing was done after each filtration cycle, using washing agents recommended by the manufacturer.

### Membrane Filtration Protocol

Before filtration, the membrane installation was flushed by circulating deionized water for 20 min and, after that, the permeation of pure water (*J*_*W*_) was measured. Filtrations were performed with a feed flow velocity of 6.8–10.3 L/min and a temperature of 20 ± 1 °C. During filtration, the concentrate was constantly circulated back to the process tank, so these values are in fact the velocities of both feed and concentrate circulating in the loop throughout the time of the process. During filtration, the time necessary for collecting each half of a liter of permeate was measured. Each permeation test was conducted until the volume of the permeate was half that of the feed solution (50% recovery and a volume concentration factor of 2). To characterize hydraulic properties of the membranes, these permeation tests were done twice for each effluent.

Based on the permeation tests, the permeate flux (filtration rate) (*J*_*V*_, Eq. 1) was calculated

1 $$ {J}_V=\frac{V_P}{t\cdot A}\;\left(\mathrm{L}/\left({\mathrm{m}}^2\kern0.5em \mathrm{h}\right)\right) $$

The recovery value (*Y*), volume concentration factor (*VCF*), and total membrane resistance (*R*_*m*_) were calculated with Eqs. 2–4

2 $$ Y=\frac{V_P}{V_F}\cdot 100\;\left(\%\right) $$

3 $$ VCF=\frac{V_F}{V_F-{V}_P}\;\left(\hbox{--} \right) $$

4 $$ {R}_m=\frac{TMP}{J_V}\;\left(\left(\mathrm{MPa}\kern0.5em \mathrm{s}\right)/\mathrm{m}\right) $$

The removal efficiency of pollutants by membranes was calculated based on the rejection coefficient (*R*, Eq. 5)

5 $$ R=\left(1-\frac{C_P}{C_F}\right)\cdot 100\;\left(\%\right) $$

The mass balance of pollutants after membrane filtration was determined with the Eq. 6

6 $$ {C}_N{V}_N={C}_P{V}_P+{C}_R{V}_R $$

The sorption abilities of the membranes were estimated as the adsorption capacity (*Ads*, Eq. 7)

7 $$ Ads=\left(1-\frac{C_R{V}_R+{C}_P{V}_P}{C_F{V}_F}\right)\cdot 100\;\left(\%\right) $$

The fouling intensity was determined by calculating the normalized permeate flux (*α*, Eq. 8)

8 $$ \alpha =\frac{J_V}{J_W}\;\left(\hbox{--} \right) $$

The abbreviations used in the equations are as follows: *A*—membrane filtration area (m^2^), *C*_*F*_—concentration of pollutants in the feed solution (mg/L), *C*_*P*_—concentration of pollutants in the permeate (mg/L), *C*_*R*_—concentration of pollutants in the retentate (mg/L), *t*—time for collecting a known volume of permeate (h), *TMP*—transmembrane pressure (MPa), *V*_*F*_—volume of feed solution (L), *V*_*P*_—volume of permeate (L), and *V*_*R*_—volume of retentate (L).

### Analytical Methods

The characteristics of the feed solution, the permeate, and the retentate were assessed in terms of TSS, COD, and BPA concentrations. TSS concentrations were measured according to APHA (1992). COD concentrations were measured with the use of LCK614 test for HACH Lange GmbH spectrophotometer. BPA concentrations were determined using an HPLC (Varian) equipped with a UV–Vis detector. A Supelcosil LC-PAH (Supelco) column was eluted with an acetonitrile/water (70:30, *v*/*v*) solution. Chromatography analysis was preceded by SPE of liquid samples using Strata X/6 mL/500 mg columns (Phenomenex). All analyses were performed in triplicate for each sample. The deviation of each measured parameter for each sample was less than 10%.

### Statistical Analyses

Differences between the samples were tested for significance using ANOVA and the Tukey’s test after normality, and homogeneity of variance was confirmed with the Shapiro-Wilk test and Levene’s test with the use of Statistica 9.0 PL (StatSoft). The strength of the relationships between groups of the results was determined using Pearson’s correlation coefficient (*r*). With all statistical analyses, *p* ≤ 0.05 was considered significant.

## Results and Discussion

The effluents from the granular sequencing batch reactors were filtered with membranes because of high concentrations of COD, suspended solids, and BPA. To find the most effective technique of filtration, both in terms of efficiency of pollutant rejection and filtration capacity, microfiltration and ultrafiltration were investigated.

**Fig. 2:**
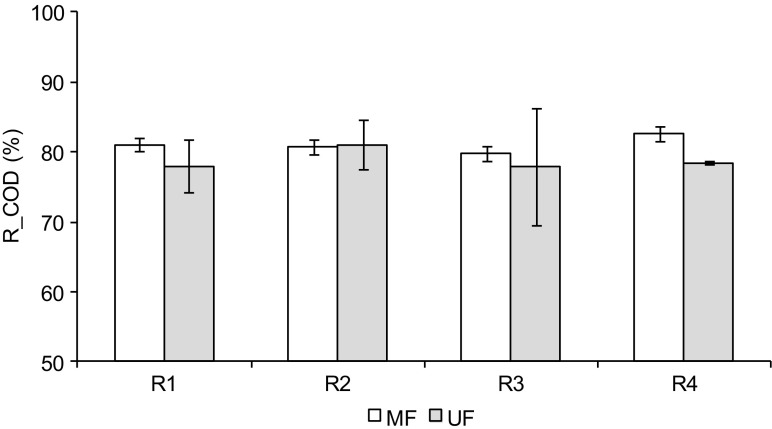
Coefficients of COD rejection (*R_COD*) in MF and UF of biologically treated wastewater from reactors *R1*–*R4*

In both MF and UF, TSS were totally removed. In the permeates, the concentrations of COD were about 24 mg/L with MF and 30 mg/L with UF, independently of the content of the feed solution. With MF, the average efficiency of COD removal, expressed as a rejection coefficient (R_COD), was from 79.8 ± 2.9% in R3 to 82.6 ± 2.8% in R4 (Fig. 2). With UF, the R_COD was from 77.9 ± 8.4% in R3 to 81.1 ± 3.5% in R2. There were no significant differences in the R_COD between the effluents from reactors R1-R4. Although the *cutoff* of the UF membrane was lower than that of the MF membrane, UF did not retain more organic compounds from the effluents from any of the reactors than MF. In both membrane techniques, sieve retention is the main mechanism responsible for removal of organic matter (Guo et al. 2009). In the present study, with MF, organic pollutants (COD) were retained at a level similar to that with UF, which indicates that COD consisted mainly of suspended particles in the examined effluents. The increase in pressure from 0.2 MPa in MF to 0.3 MPa in UF did not have any effect on retention. In all the cases, the total efficiency of COD removal in the integrated system consisting of the GSBR and membrane module ranged from 92.3 ± 0.4 to 95.5 ± 0.7%.

**Fig. 3:**
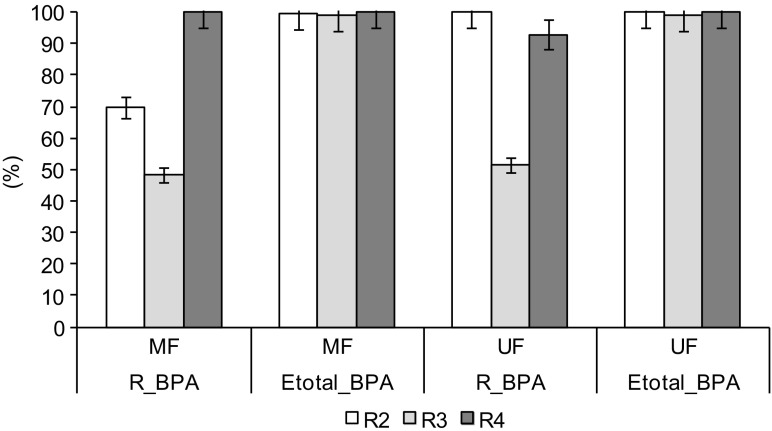
Coefficients of BPA rejection (*R_BPA*) in MF and UF of biologically treated wastewater from reactors *R2*–*R4* and total efficiency of BPA removal in the integrated technological system (*Etotal_BPA*)

Independently of the technique of membrane filtration, the total efficiency of BPA removal in the integrated system with aerobic granules and membranes exceeded 98%. After MF, BPA concentrations in the permeates equaled 0–0.052 mg BPA/L; the rejection coefficient of BPA (R_BPA) was from about 48% in R3 to 100% in R4 (Fig. 3). The permeate from UF had BPA at concentrations of 0–0.049 mg BPA/L; the R_BPA was from about 51% in R3 to 100% in R2. In general, size exclusion is the dominant mechanism for removal of large organic compounds, such as BPA, in membrane filtration. However, the pore sizes in the MF and UF membranes are several orders of magnitude larger than the BPA molecules. Therefore, high efficiency of BPA removal indicates that some of the BPA that was present in the feed solution was bound by particulate organic matter in the wastewater (suspended solids and colloids). BPA was sorbed on biomass particles; the adsorption efficiency decreased from 85 to 53% with the increasing initial BPA concentrations of 1.5–12.0 mg/L, after 24 h of sorption (data not shown). Hence, the presence of organic matter in wastewater allows BPA to be separated by membranes that are too open for the rejection of dissolved BPA.

**Fig. 4:**
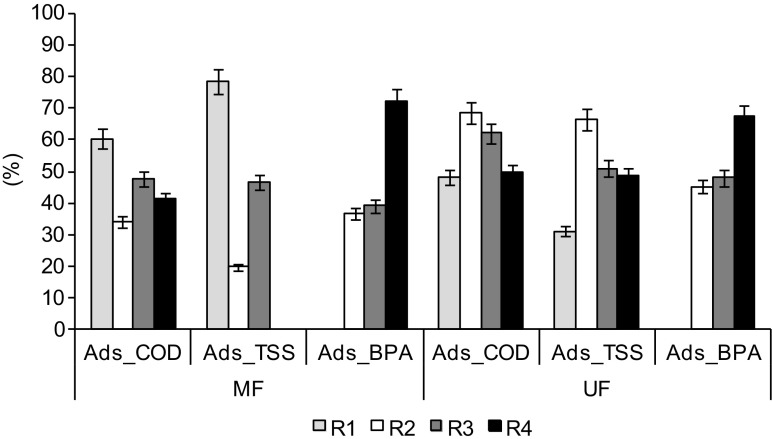
The percentage of adsorption of COD (*Ads_COD*), total suspended solids (*Ads_TSS*), and BPA (*Ads_BPA*) in MF and UF of biologically treated wastewater from reactors *R1*–*R4*

The high removal of COD and BPA, even in MF, indicates that, apart from sieve retention, adsorption on the membrane was the other mechanism responsible for organic compounds removal. The real loads of organic compounds and total suspended solids in the retentate were lower than theoretical loads that were calculated based on the mass balance (Table 2). This indicates that some part of the pollutants was sorbed on the membrane surface or captured inside the membrane structure. In UF, adsorption of COD was from 48.2 ± 2.5 to 68.6 ± 3.1%, independently of the effluent. The adsorption of TSS was from 31.1 ± 0.8 to 66.5 ± 3.4%; it was significantly higher during filtration of the effluent from R2 than during that from R1 (*p* = 0.033). In MF, adsorption of COD on the membrane was from 34.2 ± 5.4 to 60.4 ± 2.3% of total COD retention (Fig. 4). Significantly more COD was adsorbed during filtration of the effluent from R1 than during filtration of that from R2, R3, and R4 (*p* = 0.003, *p* = 0.040, *p* = 0.010, respectively), and significantly more COD was adsorbed during filtration of the effluent from R3 than that from R2 (*p* = 0.035). The adsorption of TSS ranged from 0% in R4 to 78.4 ± 3.7% in R1. Similar to COD, the adsorption of TSS during MF of the effluent from R1 was significantly higher than adsorption of that from R2, R3, and R4 (*p* = 0.001, *p* = 0.009, *p* = 0.001, respectively), it was also higher during filtration of the effluent from R3 than during filtration of that from R2 and R4 (*p* = 0.016, *p* = 0.002, respectively), and higher during filtration of the effluent from R2 than during that from R4 (*p* = 0.045). Higher adsorption during MF of the effluent from R1 may be due to higher concentrations of TSS (about 180 mg/L) than in the other effluents (about 120–150 mg/L). This higher concentrations resulted from the higher sludge yield in R1 (0.31 g MLSS/g COD versus 0.19 g MLSS/g COD in, e.g., R4). This higher sludge yield could have resulted from the fact that the presence of BPA in the influents to GSBRs inhibited COD removal and biomass growth (data not presented). In general, in MF and UF, the residual organic materials in secondary effluents, like total suspended solids, organic colloids, and exogenous polymers, are the main contributors to membrane fouling (Choo and Lee 1996; Laabs et al. 2006; Lee et al. 2006).

**Table 2:** Loads of COD and TSS (g/filtration cycle)

	Effluent from the reactor	L_F_COD_	L_P_COD_	L_R_COD_	L_exp_COD_	L_ads_COD_	L_F_TSS_	L_P_TSS_	L_R_TSS_	L_exp_TSS_	L_ads_TSS_
MF	R1	1.51	0.14	0.45	1.37	0.92	1.88	0.00	0.41	1.88	1.47
	R2	1.29	0.14	0.7	1.15	0.45	1.21	0.00	0.97	1.21	0.24
	R3	1.33	0.14	0.56	1.19	0.63	1.45	0.00	0.77	1.45	0.68
	R4	1.75	0.17	0.86	1.58	0.72	1.59	0.00	1.61	1.59	0.00
UF	R1	1.40	0.15	0.57	1.25	0.68	2.09	0.00	1.44	2.09	0.65
	R2	1.54	0.15	0.34	1.39	1.05	1.61	0.00	0.54	1.61	1.07
	R3	1.80	0.19	0.45	1.61	1.16	1.69	0.00	0.83	1.69	0.86
	R4	1.56	0.17	0.61	1.39	0.78	1.84	0.00	0.94	1.84	0.90

*L*_*F*_ measured load in the feed solution, *L*_*P*_ measured load in the permeate, *L*_*R*_ measured load in the retentate, *L*_*exp*_ expected load in the retentate (based on the mass balance), *L*_*ads*_ load that was adsorbed

The use of UF resulted in a higher percentage of COD adsorption on the membrane than use of MF, although the removal efficiencies of COD did not differ significantly. This is connected with the facts that each membrane has a specific, limited number of sorption sites and that sorption is reversible (Sun et al. 2015). The increase in the COD concentration in the feed solution correlated positively with the increase in adsorption capacity (*r* = 0.63). In MF, the increase in the concentration of TSS in the feed solution caused a significant increase in the percentage of adsorption of organic compounds and TSS on the membrane (*r* = 0.93 and *r* = 0.73, respectively). A positive correlation between percent adsorption of COD and adsorption of total suspended solids (*r* = 0.79) indicated that the lowering of COD in the permeates resulted mainly from the retention of suspended solids on the membrane surface.

In both MF and UF, the increase in BPA concentration in the feed solution caused an increase in adsorption efficiency. Removal of hydrophobic compounds, such as BPA, may proceed by their adsorption on the surface and in the inside structure of membranes (Bing-zhi et al. 2010; Escalona et al. 2014). In this study, the contribution of adsorption to the removal of BPA was estimated to be 36–72% in MF and 45–67% in UF (Fig. 4). Although the percentage of adsorption did not correlate with the efficiency of BPA removal, this removal may have been driven by adsorption on these membranes despite the large membrane pore-sizes relative to the size of the BPA molecules.

The accumulation of organic compounds on the membrane surface is considered an important mechanism of pollutant removal, but it results in membrane fouling that decreases the flux. Fouling reduces the nominal diameter of membrane pores to the so-called effective diameter, making possible the rejection of particles smaller than the membrane *cutoff* (LaPara et al. 2006; Muthukumaran et al. 2011). Fouling affects permeate flux more than pore size (Boonyaroj et al. 2012). This may explain the fairly high COD removal even in MF in this study, and the high removal of BPA in MF and UF, although the permeate fluxes did not correlate with the rejection coefficients.

In addition, the removal of BPA in membrane filtration is supposed to be affected by the fact that BPA may be sorbed on extracellular polymers that are produced by microbial cells and released from biological solids to the liquid because of shearing forces that are produced by the high-pressure pump in the membrane installation. However, because the molecular mass of EPS ranges from 31.0 to 97.4 kDa (Zhang et al. 2007), these substances are expected to appear in the permeates from both membranes. On the other hand, the fouling layer that forms on the membrane surface potentially improves rejection, leading to similar results for MF and UF membranes in this study.

**Fig. 5:**
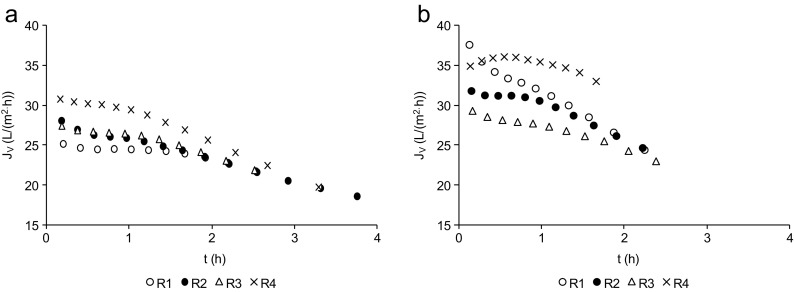
Changes in the volumetric permeate flux (*J*_*V*_) over time in MF (**a**) and UF (**b**)

The hydraulic capacity of the membrane installation was determined based on the changes in volumetric permeate flux over time (*J*_*V*_). The experiments were done with no increase in pressure to maintain a stable permeate flux; hence, *J*_*V*_ gradually decreased with the time of filtration because of blocking of the membrane with pollutants present in the feed solution. The constant circulation of the retentate to the process tank during membrane filtration causes the feed solution to constantly become more concentrated, which decreases the flux. The final measurement of permeate flux was obtained when half of the volume of the feed solution had been recovered as permeate (Fig. 5). The average permeate flux is given in Table 3. In MF, the average *J*_*V*_ in R4 was significantly higher than that in R1 (*p* = 0.016) and in R2 (*p* = 0.006). In UF, there were no significant differences between the filtrations of the effluents from the individual reactors. During filtration of the effluents from R1, R2, and R4, the use of UF resulted in significantly higher *J*_*V*_ than during MF of the effluents from those same reactors (*p* = 0.0002, *p* = 0.0002, *p* = 0.0001). In addition, the recovery of 50% was obtained after about 2.5 h of filtration in UF and about 4 h in MF (Fig. 5). This affects the frequency of membrane washing; lower frequency will lower the operational cost. The MF membrane would need higher pressure to achieve permeate flux similar to the UF membrane.

**Table 3:** Parameters of membrane filtration

	Effluent from the reactor	*J*_*V*_ (L/(m^2^ h))	*R*_*m*_ ((MPa s)/m)	*α* (–)
MF	R1	24.0	29,960	0.011
	R2	25.1	28,665	0.011
	R3	25.5	28,231	0.011
	R4	28.6	25,177	0.013
UF	R1	31.5	34,258	0.023
	R2	29.5	36,632	0.022
	R3	26.8	40,253	0.026
	R4	35.2	30,687	0.020

An increase in pressure generally increases the permeate flux. However, along with the increase in pressure, the percentage of adsorption is higher: more pollutants are retained on the membrane surface, forming a gel layer and clogging the pores. This increases filtration resistance due to the higher compression of the pollutants (Bergamasco et al. 2011; Sun et al. 2015) and, as a consequence, filtration capacity is lowered because the membrane is saturated with the pollutants. In the present study, more COD was adsorbed in UF than in MF, which resulted in higher filtration resistance in UF (Tab. 3); however, the permeate flux was still higher in UF, on average by almost 20% than in MF. Higher *R*_*m*_ in UF than in MF because of the lower *cutoff* of the UF membrane indicates that pore sizes affected the mechanism of filtration.

In this study, a large decrease of permeate flux in comparison with the flux of deionized water was observed. A value of *α* below 1 indicates that the membrane is being blocked by organic compounds that accumulate on the surface and in the pores of the membrane, which blocks the flux. This was observed in both in MF and in UF (Table 3); however, lower values of *α* for MF confirm that this membrane tended to become fouled more quickly than the UF membrane. The most important factor in membrane fouling is the ratio of the size of the pores to that of the particles (Lim and Bai 2003). Membranes with bigger pores are blocked mainly by pollutants that penetrate into the pores. In the present study, because TSS are similar in size to the membrane pores in MF, pore blockage was the main fouling mechanism, which resulted in lower permeate flux in MF than in UF. Membranes with smaller pore sizes are blocked mainly by pollutants retained on their surface, and these pollutants can be removed by shearing forces if filtration is performed in cross-flow mode, as was done in the present experiments.

## Conclusions

The results indicate that the use of ceramic membranes for MF and UF is an effective technical solution for post-treatment of effluents from reactors with aerobic granules treating wastewater that contains BPA. Independently of the membrane technique, the two-stage system of aerobic granular sludge reactor and membrane installation resulted in total removal of COD of 92–95%, TSS of 100%, and that of BPA of above 98%; however, it was achieved at a lower pressure, which could result in less consumption of energy during the filtration process. On the other hand, permeate flux averaged almost 20% higher in UF than in MF, which affects the frequency of membrane washing. These results should be taken into consideration when selecting a membrane technique for specific conditions.
